# In vitro study of elution kinetics and bio-activity of meropenem-loaded acrylic bone cement

**DOI:** 10.1007/s10195-012-0191-1

**Published:** 2012-03-30

**Authors:** Sumant Samuel, Binu S. Mathew, Balaji Veeraraghavan, Denise H. Fleming, Samuel B. Chittaranjan, John A. J. Prakash

**Affiliations:** 1 Department of Orthopedics (Unit III), Christian Medical College, Ida Scudder road, Vellore, 632004 TN India; 2 Clinical Pharmacology Unit, Christian Medical College, Ida Scudder road, Vellore, TN 632004 India; 3 Department of Clinical Microbiology, Christian Medical College, Ida Scudder road, Vellore, 632004 TN India

**Keywords:** Local antibiotic delivery, Extended-spectrum beta-lactamase producers, Gram-negative, Orthopaedic infections, Antibiotic bone cement

## Abstract

**Background:**

Use of antibiotic-loaded acrylic bone cement to treat orthopaedic infections continues to remain popular, but resistance to routinely used antibiotics has led to the search for alternative, more effective antibiotics. We studied, in vitro, the elution kinetics and bio-activity of different concentrations of meropenem-loaded acrylic bone cement.

**Methods:**

Meropenem-loaded bone cement cylinders of different concentrations were serially immersed in normal saline. Elution kinetics was studied by measuring the drug concentration in the eluate, collected at pre-determined intervals, by high-performance liquid chromatography. Bio-activity of the eluate of two different antibiotic concentrations was tested for a period of 3 weeks against each of the following organisms: *Staphylococcus aureus* ATCC 2593 (MSSA), *Enterococcus faecalis* ATCC 29212, *Pseudomonas aeruginosa* ATCC 27853, *Escherichia coli* ATCC 25922, *S. aureus* ATCC 43300 (MRSA) and *Klebsiella pneumoniae* ATCC 700603 (ESBL).

**Results:**

Meropenem elutes from acrylic bone cement for a period of 3–27 days depending on the concentration of antibiotic. Higher doses of antibiotic concentration resulted in greater elution of the antibiotic. The eluate was found to be biologically active against *S. aureus* ATCC 2593 (MSSA), *P. aeruginosa* ATCC 27853, *E. coli* ATCC 25922 and *K. pneumoniae* ATCC 700603 (ESBL) for a period of 3 weeks.

**Conclusions:**

The elution of meropenem is in keeping with typical antibiotic-loaded acrylic bone cement elution characteristics. The use of high-dose meropenem-loaded acrylic bone cement seems to be an attractive option for treatment of resistant Gram-negative orthopaedic infections but needs to be tested in vivo.

## Introduction

Multi-drug-resistant Gram-negative infections are frequently encountered in clinical practice [1]. The problem is particularly acute in Gram-negative infections due to the emergence of extended-spectrum beta-lactamase (ESBL) producers [2]. It has been recognised that Gram-negative infections are more difficult to treat than Gram-positive infections [3, 4]. Gram-negative organisms have been implicated in 10–20 % of implant-associated orthopaedic infections [5, 6]. Recent reports suggest that Gram-negative infections are emerging as a major threat in orthopaedic cases, especially in open fractures, chronic osteomyelitis, bedsores and surgical-site infections complicating internal fixation devices [7].

Acrylic bone cement (ABC) represents the current gold standard for local antibiotic delivery in orthopedic surgery, as it is a proven way to deliver high concentrations of the drug locally, especially to poorly vascularized tissues [8, 9]. Moreover, its use results in a lower serum antibiotic concentration than that associated with systemic administration, thereby reducing toxicity-related side-effects. The choice and dose of antibiotic loaded to ABC for a given situation have been the matter of much debate and research [10]. Though use of a number of antibiotics with ABC has been reported, the most widely used and studied antibiotics for this purpose are gentamicin, tobramycin and vancomycin [9, 11]. The emergence of microbial resistance to routinely used antibiotics has led to a demand for more effective antibiotics [12]. ABC containing new additives must be evaluated to ensure adequate elution from specific cement with retention of bio-activity [13].

Use of meropenem-loaded ABC has been suggested for resistant Gram-negative orthopaedic infections [14]. However, the elution kinetics and bio-activity of meropenem when loaded to ABC have not been reported. We present an in vitro study of the elution kinetics of various concentrations of meropenem-loaded ABC and test its bio-activity against micro-organisms commonly encountered in the clinical setting.

## Materials and methods

Elution kinetics of four different concentrations of meropenem-loaded surgical Simplex P^®^ bone cement cylinders (Howmedica International, Limerick, Ireland) was investigated:

- Sample A: Simplex P bone cement without meropenem (control)
- Sample B: Simplex P bone cement with 1.25 % meropenem
- Sample C: Simplex P bone cement with 2.5 % meropenem
- Sample D: Simplex P bone cement with 5 % meropenem
- Sample E: Simplex P bone cement with 10 % meropenem

Antibiotic-loaded bone cement cylinders for the above-mentioned concentrations were made. Liquid monomer (5 ml) was added to methylmethacrylate powder (10 g) in an inert bowl as per the manufacturer’s instructions. At the early ‘dough’ phase, immediately after wetting the cement, meropenem (Meronem^®^; AstraZeneca, UK) of appropriate weight to achieve the desired concentration was added and thoroughly mixed with the cement mixture in a standard fashion of one revolution per second to obtain a homogeneous compound. Cylinders of antibiotic bone cement for each concentration were made in a standardized fashion using non-expansible inert plastic tube moulds. The cylinders closely resembled the antibiotic beads used in practice and measured 16 mm in length and 12 mm in diameter. The exact weight of the cylinders was measured. The entire process was done in strict aseptic conditions.

Three cylinders of each concentration of antibiotic bone cement (samples B, C, D and E) and one control cylinder of bone cement without antibiotic (sample A) with no visible imperfections were immersed separately in 30 ml saline solution in sterile containers maintained at 37 °C without stirring and protected from light. At fixed times, after the containers were vortexed for 1 min, aliquots of 1 ml solution from each container were transferred into polypropylene test tubes for analysis. The cylinders were then rinsed in 10 ml saline solution and transferred to a new container with 30 ml saline solution at 37 °C. Sampling was similarly performed at 1, 2, 4, 8 and 24 h after immersion, then every 24 h for the next 2 days, at day 6 and finally once a week for a further 4 weeks (final sampling at 34 days after immersion).

### HPLC assay for the measurement of meropenem

Samples were assayed by isocratic high-performance liquid chromatography (HPLC) with ultraviolet (UV) detection. The mobile phase was 10 % acetonitrile and 90 % ammonium acetate buffer (50 mM, pH 5.0) at rate of 1 ml/min. The analytical column was a Supelco(discovery) HS F5, 5 μm, (250 × 4.6 mm). Detection was at 295 nm, and the temperature was maintained at 30 °C. The run time was 12 min, and there were no interferences detected from samples withdrawn from bone cement cylinders (without antibiotic) in normal saline. The minimum detectable concentration was 0.1 μg/ml. The intraday coefficient of variation for std 1 μg/ml and 100 μg/ml was 3.0 and 1.7 %, respectively. The interday quality control coefficient of variation was 3.7 %. Samples collected from the bone cement cylinders were analysed on the day of collection. The total amount of antibiotic released by each cylinder at each time point was obtained by multiplying the concentration (μg/ml) by 30, the total volume (ml) of saline in which it had been immersed. The elution rate (μg/h) of each cylinder was obtained by dividing the total quantity of antibiotic released by the elution time (in hours) of each interval. For each sample, the elution rate at different time points was plotted on a logarithmic scale. The log scale transformed rates were compared between the four groups using one-way analysis of variance (ANOVA).

### Bio-activity of meropenem

Bio-activity of two different antibiotic concentrations (samples D and E) was tested for a period of 3 weeks against each of the following organisms: *Staphylococcus aureus* ATCC 2593 (MSSA), *Enterococcus faecalis* ATCC 29212, *Pseudomonas aeruginosa* ATCC 27853, *Escherichia coli* ATCC 25922, *S. aureus* ATCC 43300 (MRSA) and *Klebsiella pneumoniae* ATCC 700603 (ESBL). Six antibiotic cylinders were prepared for each of the two tested antibiotic concentrations, 5 % (sample D) and 10 % (sample E), as described above. Cylinders without added antibiotic (sample A) served as controls.

Culture media with bacterial concentration of 10^5^ colony-forming units (CFU)/ml were created for each strain. The cylinders were immersed in 10 ml of this culture media. Ten microlitres of medium was collected at 24 and 48 h and day 7, and used to seed Mueller–Hinton agar plates. At 24 h from sampling, the bacterial culture count was taken. Each cylinder was then rinsed in 10 ml sterile physiological saline and immersed in a fresh solution of culture media with 10^5^ CFU of the same micro-organism. Samples were collected at day 14 and subcultured. The procedure was then repeated, and final sampling was done on day 21. The procedure was discontinued for micro-organisms that exhibited growth at day 7, as this indicated lack of susceptibility of the micro-organism to that drug.

## Results

### Elution kinetics

**Fig. 1:**
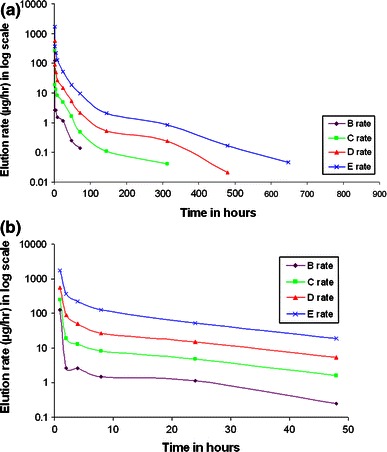
**a** Elution rate on log scale for the different samples at the measured time-points. **b** Elution rate on log scale for the different samples at the measured time-points over the first 48 h

The mean concentration of eluted meropenem for samples B, C, D and E at different time-points is presented in Table 1. The duration of antibiotic elution varied depending on the concentration of the antibiotic added. Sample E with 10 % antibiotic concentration eluted for the longest period (27 days). Figure 1a, b shows the elution rate in μg/h at different time points plotted on a logarithmic scale. All samples showed high early release rates followed by rapid decay, in keeping with typical antibiotic-loaded ABC elution characteristics. Analysis of rates (by ANOVA) revealed that there was a significant difference between the mean log elution rate of sample B and E at time-points of 8 h (*p* = 0.016), 24 h (*p* = 0.005), 36 h (*p* = 0.005), 72 h (*p* = 0.004) and 144 h (*p* = 0.025). There was no significant difference in elution rate between other samples at these time-points. Also there was no significant difference in the elution rate for any of the samples at the time-points before 8 h and after 144 h.

**Table 1:** Mean ± standard deviation concentration of meropenem (in μg/ml) of four samples

Time (h)	Sample B	Sample C	Sample D	Sample E
1	4.17 ± 0.33	8.50 ± 1.53	18.72 ± 1.26	57.83 ± 7.45
2	0.09 ± 0.15	0.63 ± 0.37	2.98 ± 0.57	12.11 ± 2.43
4	0.17 ± 0.15	0.88 ± 0.43	3.35 ± 0.21	14.83 ± 0.86
8	0.20 ± 0.17	1.08 ± 0.51	3.52 ± 0.84	16.97 ± 1.45
24	0.60 ± 0.21	2.58 ± 0.93	8.01 ± 1.07	27.94 ± 3.67
48	0.20 ± 0.17	1.30 ± 0.67	4.30 ± 0.58	15.19 ± 3.56
72	0.11 ± 0.00	0.39 ± 0.19	1.72 ± 0.19	7.64 ± 1.83
144	0.00	0.26 ± 0.00	1.25 ± 0.21	5.07 ± 0.64
312	0.00	0.23 ± 0.00	1.34 ± 0.20	4.68 ± 0.19
480	0.00	0.00	0.12 ± 0.00	0.93 ± 0.17
648	0.00	0.00	0.00	0.26 ± 0.00
816	0.00	0.00	0.00	0.00

**Fig. 2:**
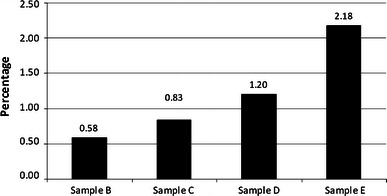
The percentage of total antibiotic released from the four samples

Figure 2 shows the percentage of antibiotic released. Higher antibiotic concentrations resulted in greater elution of the antibiotic incorporated. The total antibiotic eluted from sample C was 30 % more than for sample B. Likewise, sample D eluted 31 % more than sample C, and sample E eluted 44.7 % more than sample D.

### Bio-activity of meropenem

The eluate from sample A (control) did not show any anti-microbial activity. The results of bio-activity of the eluate of samples D and E are given in Table 2. *Enterococcus faecalis* ATCC 29212 and *Staphylococcus aureus* ATCC 43300 (MRSA) were resistant to the eluate of both samples at day 7, and further testing was discontinued. The eluate of both samples were active against the other tested micro-organisms, *Staphylococcus aureus* ATCC 2593 (MSSA), *P. aeruginosa* ATCC 27853, *E. coli* ATCC 25922 and *K. pneumoniae* ATCC 700603 (ESBL) for a period of 3 weeks.

**Table 2:** Bio-activity of samples D and E

Sample	Time									
	24 h		48 h		7th day		14th day		21th day	
	D	E	D	E	D	E	D	E	D	E
*Klebsiella*										
ATCC 700603	>10^4^ <10^5^	>10^4^ <10^5^	10^3^	10^4^	NG	NG	NG	NG	NG	NG
*E. coli* ATCC 25922	>10^5^	10^4^	10^3^	10^3^	NG	NG	NG	NG	NG	NG
*E. faecalis*										
ATCC 29212	>10^5^	>10^5^	>10^5^	>10^5^	>10^5^	>10^5^	ND	ND	ND	ND
MRSA										
ATCC 43300	>10^5^	>10^5^	10^5^	10^5^	10^5^	10^5^	ND	ND	ND	ND
MSSA										
ATCC 25923	>10^5^	>10^5^	>10^5^	10^3^	NG	NG	NG	NG	NG	NG
*P. aeruginosa*										
ATCC 27853	>10^5^	>10^5^	10^5^	>10^4^ <10^5^	NG	NG	NG	NG	NG	NG

NG, no growth; ND, not done

## Discussion

The concept of local antibiotic delivery by antibiotic-loaded acrylic bone cement was introduced by Buchholz and Engelbrecht in 1970 [15]. Since then, its clinical applications have been directed towards treatment of osteomyelitis and prosthesis infections [8, 10]. Self-mixed antibiotic ABC beads are routinely used in clinical practice as they are readily available, cheap and antibiotic specific [16].

Meropenem is a broad-spectrum bactericidal agent of the carbapenem family. It has a broad spectrum of in vitro activity and is effective against Gram-negative pathogens including extended-spectrum beta-lactamase (ESBL) producers and AmpC-producing Enterobacteriaceae [17]. Its use with ABC has been suggested for both prophylaxis and treatment of resistant Gram-negative infections [12, 14, 18]. Previous reported studies have only looked into the elution kinetics of vancomycin when loaded to acrylic bone cement, with and without meropenem added as a second antibiotic [12, 19]. Bio-activity of this combination has also been reported [12, 19]. The elution kinetics and bio-activity of meropenem-loaded acrylic bone cement per se have not been reported and are the subject of this study.

Our study shows that meropenem elutes in pharmacologically measurable concentrations from ABC for a period of 3–27 days depending on the quantity of antibiotic added. The characteristics of antibiotic elution from acrylic bone cement have been described in detail, showing rapid initial release which decreases exponentially with time [12]. A greater proportion of antibiotic is eluted from ABC that has a greater concentration of antibiotic. Both of these described characteristic features were seen in our study with meropenem as expected. Antibiotic elution from acrylic bone cement is mainly a surface phenomenon, and it has long been known that only a little of the added antibiotic is released. In a study similar to ours, Cerretani et al. [10] measured the release of vancomycin from cement beads that had an average weight of 8 g. Addition of 5 % vancomycin resulted in release of 6.76 mg vancomycin from Simplex bone cement (1.69 %) over a period of 5 weeks. In our study, 1.2 and 2.18 % of meropenem eluted from sample D (5 %) and sample E (10 %), respectively, over a period of 5 weeks. Our study also shows that the eluate is biologically active against *Staphylococcus aureus* (MSSA), *P. aeruginosa*, *E. coli* and *K. pneumoniae* (ESBL) for a period of at least 3 weeks. The spectrum of antibacterial activity is similar to established in vivo pattern.

The optimum dosing of antibiotics in bone cement with regards to safety and efficacy has yet to be determined [20]. Higher antibiotic doses are recommended when the indication is therapeutic, which is the case when acrylic cement is used as beads or spacers [20]. Hanssen classified antibiotic-loaded bone cement into high dose (>2 g antibiotic per 40 g cement) and low dose (<2 g antibiotic per 40 g cement) and recommended high dose for use as beads or spacers and low dose for prosthesis fixation [21]. It is postulated that mixing high doses of powdered antibiotics leads to increased elution of antibiotics due to increase in cement porosity [11, 20]. In this study the use of 5 and 10 % meropenem concentrations led to elution of the antibiotic for a period of 20 and 27 days, respectively.

Use of meropenem-loaded ABC for prophylaxis in arthroplasty has been suggested. For infection prophylaxis, a dose of 1.25 % meropenem with 1.25 % vancomycin has been suggested as a compromise between antibacterial properties and preservation of mechanical strength [18]. In our study we found that, at this low dose, meropenem elutes for only a period of 3 days. It is however possible that, when used with vancomycin as suggested, elution of meropenem would be enhanced due to the well-described phenomenon of ‘passive opportunism’ [18].

Gram-negative infections have traditionally been recognised as the most difficult to treat [4]. Recent literature suggests that an increasing number of Gram-negative organisms are now ESBL producers, further complicating treatment of these infections [2]. Meropenem has proven to be one of the most effective antibiotics for this class of organisms [22]. High-dose meropenem-loaded ABC may prove to be an invaluable tool against this difficult-to-treat class of orthopaedic infections. Our study has certain limitations. First, though different doses of meropenem were tested, only one type of bone cement and method of preparation was chosen for the study. However, as all the specimens were prepared and tested in a uniform and reproducible manner, we believe that these results provide useful information. The second limitation is that this is an in vitro study under laboratory conditions, and it is well recognised that there is a considerable difference between in vitro and in vivo parameters. Elution characteristics in a clinical wound may not be consistent with in vitro data [23]. Therefore, in vivo tests which emulate desired clinical performance are required to confirm the clinical efficacy of the presented data.
